# Adjuvant Treatment with Xiaoqinglong Formula for Bronchial Asthma in Acute Attack: A Systematic Review of Randomized Controlled Trials

**DOI:** 10.1155/2020/8468219

**Published:** 2020-09-14

**Authors:** Long Wang, Xianrong Feng, Baojia Wang, Yu Yang, Tianyao Zhang, Xiaobo Zhang

**Affiliations:** ^1^School of Basic Medicine, Chengdu University of Traditional Chinese Medicine, Chengdu, Sichuan 610072, China; ^2^Hospital of Chengdu University of Traditional Chinese Medicine, Chengdu, Sichuan 610075, China

## Abstract

**Background:**

XQLF (Xiaoqinglong formula) is the most commonly used prescription of traditional Chinese medicine in the treatment of asthma. XQLF combined with western medicine has been used to treat bronchial asthma in more and more cases, and good results have been achieved. Therefore, this meta-analysis aimed to evaluate the adjuvant treatment of traditional Chinese medicine classic herbal formula XQLF with bronchial asthma in acute attack.

**Methods:**

The following electronic databases were systematically searched from inception to April 2019: PubMed, EMBASE database, Cochrane Library, China National Knowledge Infrastructure (CNKI), WanFang, VIP Database for Chinese Technical Periodicals, and China Biology Medicine (CBM). Two reviewers searched these databases and independently evaluated all the eligible articles for inclusion. Stata 14.0 was used for data synthesis and analysis.

**Results:**

A total of 33 RCTs (randomized controlled trials) including 2176 patients were enrolled. All of the patients in these studies were in the acute attack stage of asthma. We conducted subgroup analysis according to the duration of treatment, which was 14 days, 10 days, and 7 days, respectively. The overall results show that adjuvant treatment with XQLF significantly improve CER (clinical efficacy rate) (RR = 1.17; 95% CI, 1.14 to 1.21; *P* < 0.0001) and promote pulmonary function including FEV1 (WMD = 0.35; 95% CI, 0.27 to 0.43; *P* < 0.0001), PEF (SMD = 1.02; 95% CI, 0.49 to 1.55; *P* < 0.0001), and FVC (WMD = 0.51; 95% CI, 0.35 to 0.66; *P* < 0.0001). The adjuvant treatment of XQLF can also reduce serum IgE concentration (SMD = −1.39; 95% CI, 1.92 to −0.85; *P* < 0.0001) and serum EOS concentration at 14 days (WMD = −39.85; 95% CI, −56.20 to −23.49; *P* < 0.0001).

**Conclusion:**

This study finally showed that XQLF has the auxiliary effect of improving the efficiency, promoting the lung function, and reducing the serum IgE in the treatment of acute attack asthma. This trial is registered with CRD42019133549.

## 1. Introduction

Bronchial asthma is one of the most common chronic diseases in the world and has become one of the reasons that seriously affect public health [1]. Bronchial asthma is a chronic inflammatory disease of the airway, which involves a variety of cellular and cellular components [1, 2]. This chronic inflammation can lead to airway hyperresponsiveness, widespread and variable reversible airflow limitation, and cause recurrent wheezing, shortness of breath, chest tightness, or cough [3]. Acute attack stage is relatively dangerous for patients with bronchial asthma, which can lead to respiratory dysfunction, such as dyspnea and chest tightness. If the treatment is not timely, patients may even die. Therefore, the treatment of acute attack stage of asthma should aim at quickly alleviating symptoms, improving hypoxia and regulating pulmonary function [4]. The complexity of asthma pathogenesis leads to the complexity of asthma treatment. At present, drug control is still the main treatment for asthma. Although there are many kinds of drugs available in clinic, regular inhalation of glucocorticoid is still the basic drug for maintenance treatment and control of asthma. However, many patients cannot control asthma by using low-dose glucocorticoid, and high-dose glucocorticoid can produce serious side effects. Therefore, the combination of drugs began to be considered. The current research focuses on how to choose the drug types and administration methods reasonably. For example, long acting *β*2-receptor agonist combined with low-dose inhaled glucocorticoid can effectively alleviate the clinical symptoms of asthma patients, improve the lung function, reduce the frequency of asthma attack, and also reduce the dosage of short acting *β*2-receptor agonist [5]. There are also studies that show that leukotriene modifiers as an adjuvant can effectively reduce the dose of inhaled glucocorticoids in patients with moderate and severe asthma [6, 7]. In addition to the combined use of Western medicine, traditional Chinese medicine as an adjuvant therapy was also considered. In China, more and more clinical reports showed that combination of Chinese and Western medicines could quickly and effectively alleviate bronchial in acute attack.

XQLF is the most commonly used prescription of traditional Chinese medicine in the treatment of asthma. In recent years, many studies have shown that this prescription can relieve cough and asthma, and it also has antipyretic, anti-inflammatory, and antiallergic effects. There are many dosage forms of XQLF in clinic, such as decoction, mixture, and granule, but decoction is the most widely used. In China, XQLF combined with western medicine has been used to treat bronchial asthma in more and more cases, and good results have been achieved. For example, Diaz et al. [8] found that the effects of fluticasone inhalation combined with XQLD (Xiaoqinglong decoction) on pulmonary function and serum IL-16 levels were superior to those of fluticasone inhalation and XQLD alone in asthma patients. Zha et al. [9] also found latent class analysis of Chinese medicine symptom patterns was able to define a subset of patients who would respond to XQLG (Xiaoqinglong granules) add-on therapy significantly better than placebo. Therefore, this article intends to systematically evaluate the auxiliary role of XQLF in the treatment of bronchial asthma in acute attack.

## 2. Methods

This systematic review and meta-analysis was based on a prespecified protocol and was reported according to the preferred reporting items for systematic reviews and meta-analyses (PRISMA) statement. We have registered the protocol on the PROSPERO, and the number is CRD42019133549.

### 2.1. Search Strategy and Selection Criteria

This meta-analysis was conducted according to the recommendations of the Preferred Reporting Items for Systematic Reviews and Meta-analyses Statement [10]. The following electronic databases should be systematically searched from inception to April 2019: PubMed, EMBASE database, Cochrane Library, CNKI, Wan Fang, VIP Database for Chinese Technical Periodicals and China Biology Medicine disc, and CBM. In addition, we also manually searched additional relevant studies, using references from systematic reviews published previously. The search terms were “Traditional Chinese Medicine” and “Combination of Traditional Chinese and Western Medicine,” “xiaoqinglong,” “xiaoqinglongtang,” “XQLF,” “XQL,” “asthma,” “bronchial asthma,” “randomized controlled trials.” Each search term was retrieved separately or in combination. All databases were retrieved from self-built databases until April 15, 2019. In order to prevent missing search and expand the search scope, we did not search for the term “acute attack.”

### 2.2. Inclusion Criteria

(1) RCT: any reference to “random grouping” or “random sequence” in the study should be deemed as RCT, whether single blind, double blind, or no blind, and the language was limited to Chinese and English. (2) Types of participants: patients with lung cancer, bronchiectasis, pneumonia, chronic bronchitis, and other lung diseases were excluded. Asthma patients in the acute attack stage, regardless of age and duration of disease. Patients in remission stage, persistent state, and severe and occupational asthma were excluded. (3) Intervention measures: the control group was treated with conventional western medicine, including bronchodilator, expectorant, glucocorticoid, *β*2-receptor agonist, and sustained-release theophylline. The experimental group was treated with XQLF on the basis of conventional western medicine. XQLF, regardless of dosage form (tablet, mixture, and decoction), must contain the original drug composition. The treatment time of both groups was less than or equal to 14 days. (4) Outcomes: the primary outcomes included clinical efficacy rate and pulmonary function. Clinical effectiveness included clinical control and effective and significant effect. Significant efficiency: the symptoms and signs related to asthma were significantly improved or completely disappeared, occasionally with mild attack but rapid relief and accompanied by the disappearance of wheezing sound; effective: asthma-related symptoms and signs were improved, and attack duration was shortened and wheezing rale was reduced; no effect: asthma-related symptoms and signs were not significantly improved or worsened; total effective rate (%) = effective rate (%) + significant efficiency rate (%). pulmonary function. It mainly includes 3 outcomes to evaluate lung function comprehensively, which are FEV1, PEF, and FVC. The secondary outcomes were EOS and IgE. EOS and IgE are important reference indexes in the diagnosis and treatment of bronchial asthma.

### 2.3. Data Extraction and Quality Evaluation

Literature retrieval, study inclusion, and data extraction were carried out independently by two authors (Xian-Rong Feng and Xiao-Bo Zhang), and disagreements were resolved by consensus or arbitrated by a third author (Yu Yang). Standard data extraction tables were used to extract relevant information and data. The extracted content includes the author's name, publication time, and sample size of each clinical study. The age, gender, intervention methods, outcomes, and adverse reactions of participants should also be included. If the information provided by the study was incomplete, try to contact the author for access. If it was still not available, the study should be excluded.

The methodological quality of the included trials should be evaluated using the *Cochrane Handbook for Systematic Reviews of Interventions*. It mainly includes the following: selection bias (random sequence generation and allocation concealment), performance bias (blinding of participants and personnel), detection bias (blinding of outcome assessment), attrition bias (incomplete outcome data), reporting bias (selective reporting), and other sources of bias. Quality of each item will be divided into low/unclear/high risk of bias. Any disagreements will be analyzed by the third reviewer.

### 2.4. Data Synthesis and Analysis

RR, WMD, and 95% confidence intervals (CI) were calculated for dichotomous data and continuous data, respectively. If the units of continuous variable data are not uniform, then SMD is used as the effect size. Statistical heterogeneity among studies was assessed using the *I*^2^ statistic and the Cochrane Q statistic. Data were analyzed with a fixed-effect model if no statistical heterogeneity was indicated (*I*^2^ < 50% or *P* > 0.10) [10], and if statistical heterogeneity was indicated (*I*^2^ > 50% or *P* < 0.10), pooled effect sizes were calculated by the random-effects model. If there were more than 10 eligible trials included in the meta-analysis for a specific outcome, publication bias was assessed by constructing a funnel plot. Publication bias was also evaluated by Egger's test, and a significant publication bias was defined as a *P* value <0.1. Subgroup analyses were conducted, if the heterogeneity is still very high after using the random effect model. The STATA 14.0 software was used for data synthesis and analysis. A *P* value less than 0.05 was considered as statistically significant in the pooling results.

## 3. Result

### 3.1. Study Selection

**Figure 1:**
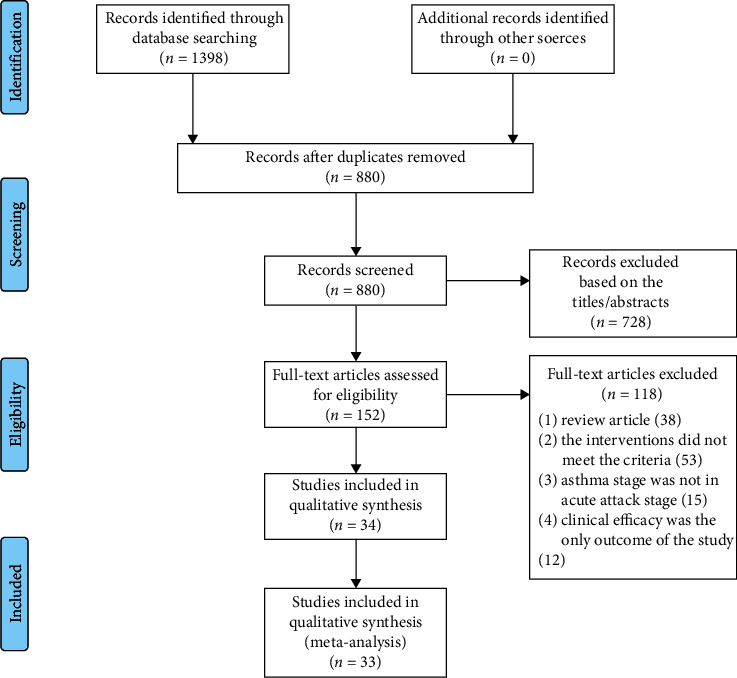
Flow chart of selection process.

From the electronic database, we have retrieved a total of 1398 original studies. After the deletion of duplicate studies, 880 studies remained. After that, we selected 152 studies by reading the titles and abstracts of the articles. Through careful review of the inclusion criteria and assessed full-text articles, 118 studies were excluded again. The reasons for exclusion include the following: review article (38); control group was not conventional western medicine treatment (31); experimental group had other traditional Chinese medicine treatment measures (22); asthma stage was not in acute attack stage (15); clinical efficacy was the only outcome of the study (12). In the remaining 34 studies, it was found that the outcomes in one study did not have clear unit and did not meet the inclusion criteria, and 33 studies were included to analyze the results eventually (Figure 1).

### 3.2. Characteristics of Included Studies

A total of 33 RCTs including 2176 patients were enrolled, among which 1328 were in experimental group and 1320 in the control group. All of the patients in these studies were in the acute attack stage of asthma. Specific interventions were 12 studies using the original XQLF decoction combined with conventional treatment compared with conventional treatment alone, 16 studies using the modified XQLF decoction combined with conventional treatment, 4 studies for XQLF granules, and one study for XQLF mixture. Of the study population, 4 were children and 29 were adults. Only 1 study mentioned the occurrence of adverse reactions [11]. The treatment duration of 18 trials lasted up to 14 d, 5 trials lasted up to 10 d, and 13 studies lasted up to 7 d. The summary characteristics of the included studies are shown in Table 1.

## 4. Methodological Quality

**Figure 2:**
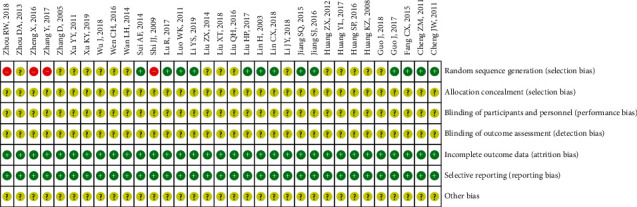
Risk of bias summary. Dash sign: high risk of bias; plus sign: low risk of bias; question mark sign: unclear risk of bias.

The final 34 studies all claimed that they were randomized controlled trials. Among them, 13 RCTs [12–24] explicitly indicate that random number table method was used to realize randomization, 3 RCTs [25–27] described that they used the method of admission sequence to achieve random grouping, and 1 RCT [28] used odd even number to realize randomization. The rest of RCTs did not clearly describe the randomization. Allocation concealment and blinding were not addressed in all studies. We utilized the criteria recommended by Cochrane Handbook for Systematic Reviews to assess the risk of bias in the 33 articles included. A risk of bias summary is shown in (Figure 2).

### 4.1. Meta-Analysis of Measured Outcomes

**Figure 3:**
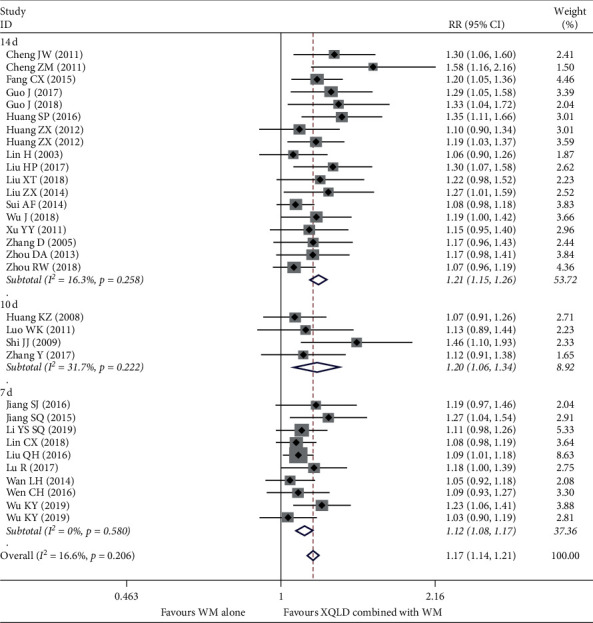
Forest plot of the comparison between combined treatment group and the control group for the outcome CER. The subgroup analysis was performed based on different treatment durations: 7 d, 10 d, and 14 d.

A total of 31 studies reported clinical effective rate including 2563 patients. We conducted subgroup analysis according to the duration of treatment, which was 14 days, 10 days, and 7 days, respectively. The overall pooled result yielded a statistically significant increase in CER in the combination treatment group, which indicated that CER in the combined treatment group was 1.17 times of that in the control group (RR = 1.17; 95% CI, 1.14–1.21; *P* < 0.0001). A mild heterogeneity presented between studies (*I*^2^ = 16.6%, *P*=0.206). In addition, we found that with the increase of treatment time, the clinical effective rate was also increased, and the pooled result was 7 days (RR = 1.12; 95% CI, 1.08–1.17; *P* < 0.0001), 10 days (RR = 1.20; 95% CI, 1.06–1.34; *P* < 0.0001), and 14 days (RR = 1.21; 95% CI, 1.15 to 1.26; *P* < 0.0001), respectively, and the result is shown in (Figure 3).

### 4.2. Pulmonary Function

#### 4.2.1. FEV1

**Figure 4:**
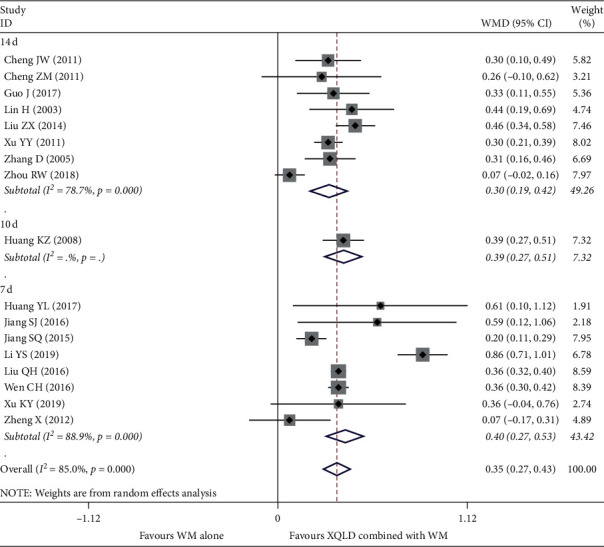
Forest plot of the comparison between combined treatment group and the control group for the outcome FEV1. The subgroup analysis was performed based on different treatment durations: 7 d, 10 d, and 14 d.

17 studies [12, 13, 15–18, 20, 25, 27–36] reported FEV1, with a total of 1504 patients. Since the pooled results of analyzing with fixed-effect model show high heterogeneity among the studies, the random effect model is used. Pooled analysis showed that combination use of XQLF and conventional treatment had certain superiority in increasing the FEV1 over conventional treatment alone (WMD = 0.35; 95% CI, 0.27 to 0.43; *P* < 0.0001). However, there were high heterogeneity among the studies (*I*^2^ = 85%, *P* < 0.0001). After subgroup analysis with different treatment time, there was still relatively high heterogeneity: 14 d (*I*^2^ = 78.7%, *P* < 0.0001) and 7 d (*I*^2^ = 88.9%, *P* < 0.0001); the result is shown in (Figure 4).

#### 4.2.2. PEF

**Figure 5:**
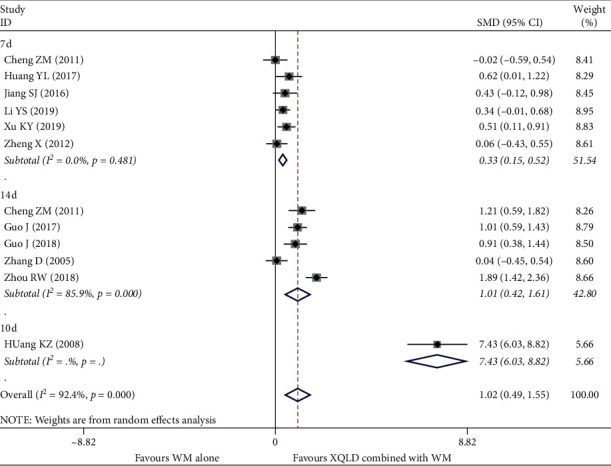
Forest plot of the comparison between combined treatment group and the control group for the outcome PEF. The subgroup analysis was performed based on different treatment durations: 7 d, 10 d, and 14 d.

11 studies [13, 15, 16, 25, 27–30, 34, 36, 37] reported PEF including a total of 875 patients. PEF values in these studies were not in the same unit and cannot be converted, so SMD was used for the synthesis of its effect size. After using the random effect model, the pooled results showed that the combination group was better than the conventional treatment group in improving PEF (SMD = 1.02; 95% CI, 0.49 to 1.55; *P* < 0.0001). But, the heterogeneity between the studies was high (*I*^2^ = 92.4%, *P* < 0.0001). After subgroup analysis, the heterogeneity was 7 d (*I*^2^ = 0.0%, *P*=0.481) and 14 d (*I*^2^ = 85.9%, *P* < 0.0001), respectively; the result is shown in (Figure 5).

#### 4.2.3. FVC

**Figure 6:**
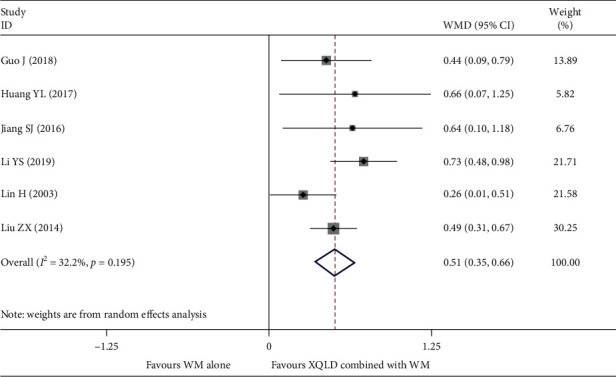
Forest plot of the comparison between combined treatment group and the control group for the outcome FVC.

Six studies [16, 18, 20, 30, 32, 37] reported FVC, with a total of 403 patients. After using the random effect model, the pooled results showed that the combination group had better effect on improving FVC than the conventional treatment group (WMD = 0.51; 95% CI, 0.35 to 0.66; *P* < 0.0001) and low heterogeneity between studies (*I*^2^ = 32.2%, *P*=0.195). The results are shown in (Figure 6).

#### 4.2.4. Ig E

**Figure 7:**
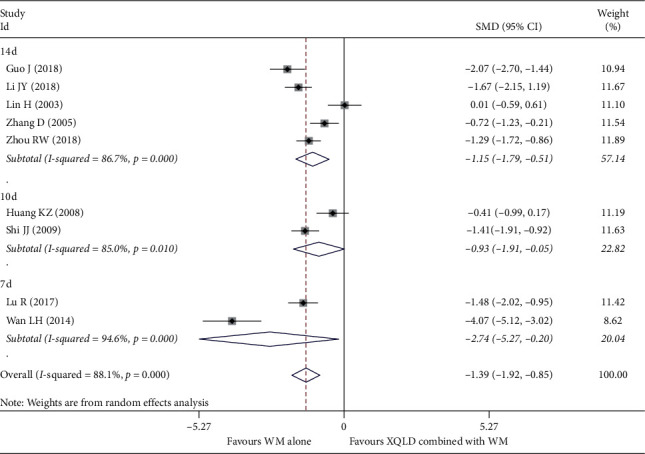
Forest plot of the comparison between combined treatment group and the control group for the outcome IgE. The subgroup analysis was performed based on different treatment durations: 7 d, 10 d, and14 d.

Because the units of Ig E in each study are not uniform and cannot be converted, SMD is used to synthesize its effect size. 9 studies [20, 22, 26–29, 37–39] reported the concentration of serum Ig E, a total of 596 patients. After using the random effect model for analysis, the pooled results showed that the heterogeneity of each subgroup is still very high, 14 d (*I*^2^ = 86.7%, *P* < 0.0001), 10 d (*I*^2^ = 85.0%, *P* < 0.0001), and 7 d (*I*^2^ = 94.6%, *P* < 0.0001). But on the whole, pooled result shows that the combination group is more obvious in reducing the concentration of IgE compared with the control group (SMD = −1.39; 95% CI, −1.92 to −0.85; *P* < 0.0001). The results are shown in (Figure 7).

#### 4.2.5. EOS

**Figure 8:**
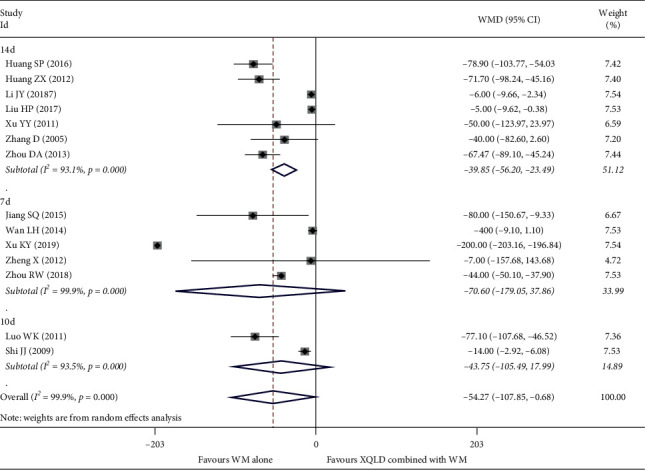
Forest plot of the comparison between combined treatment group and the control group for the outcome EOS. The subgroup analysis was performed based on different treatment durations: 7 d, 10 d, and 14 d.

14 studies [17, 21, 23, 25–28, 34, 35, 38–42] reported the serum EOS concentration for a total of 1209 patients. After using the random effect model, the pooled results show that each subgroup has high heterogeneity, 14 d (*I*^2^ = 93.1%, *P* < 0.0001), 10 d (*I*^2^ = 93.5%, *P* < 0.0001), and 7 d (*I*^2^ = 99.9%, *P* < 0.0001). There was no significant difference between the combined treatment group and the control group in the treatment of 7 d and 10 d: 7 d (WMD = −70.60; 95% CI, −179.05 to 37.86; *P*=0.202) and 10 d (WMD = −43.75; 95% CI, −105.49 to 17.99; *P*=0.165). After 14 days of treatment, compared with the control group, the combined group can significantly reduce the concentration of EOS (WMD = −39.85; 95% CI, −56.20 to −23.49; *P* < 0.0001), The results are shown in (Figure 8).

### 4.3. Publication Bias

**Figure 9:**
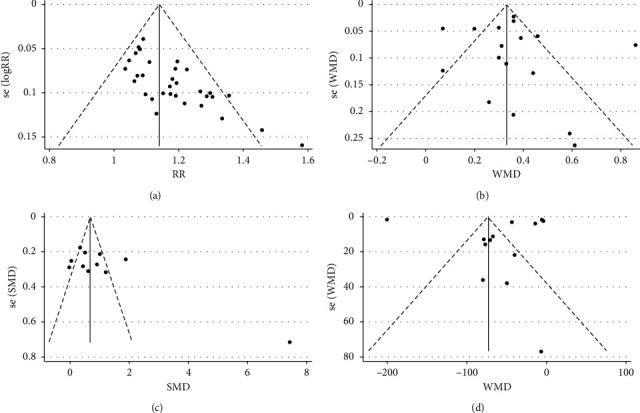
Evaluation of publication bias. Funnel plots of (a) CER, (b) FEV1, (c) PEF, and (d) EOS.

No evidence of publication bias was detected in FEV1 (Egger's test, *P*=0.712) and EOS (Egger's test, *P*=0.581). Evidence of publication bias was found in CER (Egger's test, *P* < 0.0001) and PEF (Eggers test, *P*=0.031). Publication bias was assessed by constructing a funnel plot (Figure 9).

## 5. Discussion

In recent years, the basic research on XQLF in the treatment of asthma and other respiratory diseases is gradually in-depth. For example, Song et al. [43] found XQLF may play a role in reducing inflammation and alleviating asthma by regulating the TSLP signaling pathway. However, the specific mechanism of XQLF in the treatment of asthma is still unclear. At present, there are many clinical methods for the treatment of bronchial asthma, among which the drug treatment begins to develop in the direction of combined treatment. It is necessary to consider the individual factors of patients in the selection of relevant treatment plans, which is consistent with the principle of TCM symptomatic treatment. And, this will be the focus of asthma prevention and treatment in the future. XQLF is a commonly used prescription in the treatment of bronchial asthma, which has been widely used in clinical. According to the clinical symptoms of asthma patients, the composition of XQLF is also changed in TCM treatment, which is based on the theory of symptomatic treatment. Traditional Chinese medicine believes that XQLF can improve airflow restriction and inhibit respiratory disorders and has a significant effect on the improvement of symptoms in the acute stage of asthma.

In the study selection, we excluded those studies with only CER as an outcome. Because the judgement of CER is by observing the symptoms of the patients, it has great subjectivity. Therefore, it is necessary to carry out evaluation with other objective indicators. The results of meta-analysis in our study show that XQLF combined with conventional treatment is more obvious in improving the efficiency, promoting the lung function, and reducing the Ig E compared with conventional treatment alone. In terms of CER, we found that, on the 7th, 10^th^, and 14th days of treatment, the combination group was all more significant than the conventional treatment group in improving the efficiency. Therefore, it indicated that XQLF has a very good auxiliary role in rapidly relieving clinical symptoms and improving clinical efficiency. In terms of improving the lung function, FEV1, FEF, and FVC all showed that XQLF combined with conventional treatment was superior to conventional treatment alone. After subgroup analysis of FEV1, the combined group could significantly improve FEV1 on the 7th, 10^th^, and 14th days compared with the control group. After subgroup analysis of PEF, the difference between 7-day heterogeneity and 14-day heterogeneity was very large, so we can think that the treatment duration is the main cause of heterogeneity. In addition, different methods of lung function testing may be one of the reasons for heterogeneity. However, most of the studies did not describe the testing method in detail, so it is difficult to generate subgroups for further analysis. In the results of EOS, there was no statistical difference between the combined group and the control group on the 7 days and 10 days. However, it showed statistical significance in 14 days, which may be the reason for the longer treatment time, and thus significant results were shown. In all the studies, we included XQLF including the original XQLD, the modified XQLD, and granules and mixture. We used XQLF generate subgroups for meta-analysis, and the results showed that it was not the source of heterogeneity.

A similar meta-analysis was published in China [44]. In this study, XQLF and XQLF combined with western medicine were compared with western medicine alone to observe the therapeutic effect on bronchial asthma. But, we pay more attention to the comparison between the combination of drugs and the single use of western medicine in the treatment of asthma, because considering the combination of drugs has become the first choice to control the acute attack of asthma in the clinical field. Furthermore, we focused on the acute attack stage of asthma and shortened the treatment time (<14 days) to observe the control effect on the acute attack of asthma in a short time. For the safety of combined use, only one study mentioned the occurrence of adverse reactions [11], and it is not enough for systematic evaluation.

## 6. Conclusion

The meta-analysis indicated that that XQLF has the auxiliary effect of improving the total effective rate, promoting the lung function, and reducing the serum immunoglobulin E in the treatment of acute attack asthma. However, there were some limitations in this meta-analysis, such as high clinical heterogeneity, low methodological quality of the included trials, and significant publication bias of some outcomes. Therefore, more high-quality RCTs with rigorous designing, large-scale, multicenter, and double-blinded are required to further identify the efficacy and safety of XQLF for asthma.

## Figures and Tables

**Table 1:** Characteristics of included studies.

Author	year	Number (T/C)	Gender (M/F)	Age (T/C)	Randomization	Intervention		Treatment duration (d)	Outcomes
						T/C	CT in both group		
Cheng	2011	32/35	40/27	49.95 ± 14/46.24 ± 15.4	RNT	O-XQLD (300 ml/d) + CT/CT	Budesonide powder (400∼800 *μ*g/d)	14	CER; FEV1

Cheng	2011	24/24	23/25	54.3 ± 6.4/55.6 ± 5.7	RNT	M-XQLD (1dose/d) + CT/CT	Budesonide aerosol (200–800 mg/d)	14	CER; FEV1; PEF

Fang	2015	56/56	66/46	55.1 ± 10.4/54.8 ± 10.2	RNT	M-XQLD (400 ml/d) + CT/CT	Budesonide aerosol (1600 *μ*g/d)	14	CER, SS

Guo	2017	50/50	46/54	41.61 ± 3.10/41.06 ± 3.09	RNT	M-XQLD (2dose/d) + CT/CT	Budesonide formoterol powder (160 *μ*g/d)	14	CER; FEV1; PEF

Guo	2018	30/30	Unclear	18–65	Unclear	M-XQLD(400 ml/d) + CT/CT	Budesonide formoterol powder (160 *μ*g/4.5 *μ*g)	14	FVC; PEF; Ig E

Huang	2008	32/32	37/27	42.6 ± 6.13/44.32 ± 6.18	Unclear	M-XQLD (400 ml/d) + CT/CT	Becotide aerosol (500 *μ*g/d)	10	CER; FEV1; PEF; IgE; EOS

Huang	2016	44/44	55/33	40.5 ± 10.2/39.8 ± 9.8	Unclear	O-XQLD (1dose/d) + CT/CT	Bronchodilator agent + glucocorticoid	14	CER; EOS

Huang	2017	22/22	31/13	41.24 ± 1.23/41.21 ± 1.26	Unclear	XQLG (3 bags/d) + CT/CT	Salmeterol fluticasone powder (2 dose/d)	7	FEV1; PEF; FVC

Huang	2012	39/39	33/45	39.8 ± 9.8/40.6 ± 10.3	Unclear	O-XQLD (1dose/d) + CT/CT	Bronchodilator agent + glucocorticoid	14	CER; EOS

Jiang	2016	26/26	29/23	18–65	RNT	XQLG (3bags/d) + CT/CT	Salmeterol fluticasone inhalant (100-500 *μ*g/d)	7	CER; FEV1; PEF; FVC

Jiang	2015	40/40	22/58	42.34 ± 4.21/44.34 ± 4.68	RNT	M-XQLD (100 ml/d) + CT/CT	80 mg prednisone + 5 mg ventolin (2-3 dose/d)	7	CER; FEV1; EOS

Li	2018	45/45	43/47	44.6 ± 8.7/43.6 ± 9.6	Unclear	M-XQLD (2 dose/d) + CT/CT	Aminophylline + glucocorticoid + antibiotics	14	CER; IgE; EOS

Li	2019	66/66	71/61	18–65	RNT	XQLG (3 bags/d) + CT/CT	Budesonide aerosol (2-3 dose/d)	7	CER; FEV1; PEF; FVC;

Lin	2018	40/40	43/37	40.58/40.49	RNT	O-XQLD (1 dose/d) + CT/CT	Salmeterol fluticasone inhalant (100-500 *μ*g/d)	7	CER; EOS

Lin	2003	23/20	23/20	41/40	RNT	M-XQLD (1 dose/d) + CT/CT	Receptor agonists, glucocorticoids, and antibiotics	14	CER; FEV1; FVC; Ig E

Liu	2017	36/36	39/33	29.16 ± 4.21/29.06 ± 4.35	RNT	M-XQLD (400 ml/d) + CT/CT	Bronchodilator agent + glucocorticoid	14	CER; EOS

Liu	2016	100/100	136/54	26.2 ± 3.5/25.9 ± 4.5	Unclear	O-XQLD (200 ml/d) + CT/CT	Bronchodilator agent + glucocorticoid	7	CER; FEV1;

Liu	2018	30/30	31/29	50 ± 2.1/50 ± 2.5	Unclear	M-XQLD (100 ml/d) + CT/CT	10 mg montelukast + 80 *μ*g budesonide aerosol (2 dose/d)	14	CER; AR

Liu	2014	38/38	42/34	47.9 ± 4.9	Unclear	XQLM (60 ml/d) + CT/CT	Salbutamol aerosol (0.4 mg/d)	14	CER; FEV1; FVC;

Lu	2017	35/34	43/26	9.16 ± 2.85/9.47 ± 2.41	RNT	XQLG (2 dose/d) + CT/CT	100 *μ*g fluticasone propionate + 50 *μ*g salmeterol (1 dose/d)	7	CER; Ig E

Luo	2011	30/30	27/33	40.1 ± 10.2/44.0 ± 10.2	RNT	O-XQLD (1 dose/d) + CT/CT	Bronchodilator Agent + glucocorticoid	10	CER; EOS

Shi	2009	40/40	46/34	45.3 ± 11.3/43.2 ± 11.6	Admission time	M-XQLD (unclear) + CT/CT	Theophylline + long-acting beta agonists + glucocorticoids	10	CER; IgE; EOS

Sui	2014	42/42	41/43	66.3 ± 10.46/66.26 ± 10.58	RNT	M-XQLD (300 ml/d) + CT/CT	Salbutamol aerosol (0.4 mg/d)	14	CER; SS

Wan	2014	22/22	21/23	67.62 ± 4.79/42.9 ± 9.73	Unclear	M-XQLD (1 dose/d) + CT/CT	Bronchodilator agent + theophylline + glucocorticoid	7	CER; IgE; EOS

Wen	2016	40/40	45/35	26.2 ± 3.5/25.9 ± 4.5	Unclear	O-XQLD (100 ml/d) + CT/CT	Bronchodilator agent + glucocorticoid	7	CER; FEV1;

Wu	2018	50/48	63/35	9.37 ± 1.25/8.95 ± 1.31	Unclear	O-XQLD (1dose/d) + CT/CT	Budesonide aerosol (400 *μ*g/d)	14	CER; SS

Xu	2019	50/50	44/56	52.21 ± 2.11/52.26 ± 2.15	Unclear	M-XQLD (1dose/d) + CT/CT	Salmeterol fluticasone inhalant (100–500 *μ*g/d)	7	CER; EOS; PEF; FEV1

Xu	2011	41/37	55/23	42.13 ± 8.14/41.13 ± 7.32	Unclear	O-XQLD (unclear) + CT/CT	Drugs of relieving cough and subsiding wheeze	14	CER; EOS; FEV1

Zhang	2005	33/30	37/26	3 months to 14 years old	Unclear	M-XQLD (1 dose/d) + CT/CT	Hydrocortisone succinate: 10 mg/(kg d) (3 doses/d)	14	CER; FEV1; PEF; EOS; IgE

Zhang	2017	20/20	24/16	43.5 ± 9.7/44.6 ± 10.8	Admission time	O-XQLD (300 ml/d) + CT/CT	Montelukas (10 mg/d)	10	CER; FEV1

Zheng	2012	32/32	29/35	45.3 ± 8.7/42.9 ± 9.1	Admission time	O-XQLD (300 ml/d) + CT/CT	Salmeterol fluticasone inhalant + salbutamol	7	CER; FEV1; PEF; EOS

Zhou	2013	52/53	39/66	41.95 ± 8.3/40.31 ± 6.9	Unclear	M-XQLD9unclear) + CT/CT	Bronchodilator agent + glucocorticoid	14	CER; EOS

Zhou	2018	50/50	53/47	41.7 ± 14.17/42.82 ± 13.58	Odd even number	O-XQLD (1 dose/d) + CT/CT	Budesonide nasal spraying agent (200 *μ*g/d)	14	CER; FEV1; PEF; IgE; EOS

AR: adverse reactions; C: control group; CER: clinical efficiency rate; CT: conventional treatment; d: days; M-XQLD: modified xiaoqinglong decoction; O-XQLD: original xiaoqinglong decoction; RNT: random number table; SS: symptom scores; T: treatment group; XQL: xiaoqinglong; XQLG: xiaoqinglong granules; XQLM: xiaoqnglong mixture.

## Data Availability

All data generated or analyzed during this study are included in this published article.

## Abbreviations

CNKI: China National Knowledge Infrastructure
CBM: China Biology Medicine
XQLF: Xiaoqinglong formula
RCT: Randomized controlled trial
CER: Clinical efficacy rate
XQLD: Xiaoqinglong decoction
XQLG: Xiaoqinglong granules
TCM: Traditional Chinese Medicine.
